# Recognition and inequalities in older adults' sexuality in Chile

**DOI:** 10.3389/fsoc.2024.1368104

**Published:** 2024-04-10

**Authors:** Verónica Gómez-Urrutia, Andrea Gartenlaub, Felipe Tello-Navarro

**Affiliations:** ^1^Facultad de Ciencias Sociales y Humanidades, Universidad Autónoma de Chile, Talca, Chile; ^2^Facultad de Comunicaciones y Artes, Universidad de Las Américas, Santiago, Chile; ^3^Facultad de Ciencias Sociales y Comunicaciones, Universidad Santo Tomás, Talca, Chile

**Keywords:** aging, sexuality, gender, class, perceptions

## Abstract

**Introduction:**

This paper explores older adults' perceptions on sexuality and affectivity in Chile, according to class and sex.

**Methods:**

The study is based on computer-assisted telephonic interviews with people aged 60 and over, men and women (*n* = 481). Data were analyzed using chi-squared tests and binary logistic regressions.

**Results and discussion:**

Maintaining an active sex life is important for older adults of both sexes, contradicting the commonsense view according to which the relevance allocated to sex decreases significantly with age. However, the data show significant differences in perceptions by sex, suggesting that gendered conceptions regarding sexuality are influential along the entire life cycle. There are also relevant differences according to class, revealing the inequalities present in the expression of sexuality in Chile.

## 1 Introduction

Until quite recently, older age has been associated with asexuality (Hinchliff and Gott, 2016; Curley and Johnson, 2022; Cameron and Santos-Iglesias, 2024). However, there is a growing interest in moving beyond this stereotype toward the idea that sexuality is very important throughout life, although the way it is experienced changes (Carpenter, 2015; Gore-Gorszewska et al., 2023). Contrary to the rather negative view on aging as a loss -of vitality, attractiveness, and sex drive- it is now understood that the physical changes that occur as part of the aging process might change how people experience sexuality, but do not necessarily reduce its centrality to individuals' wellbeing (Gewirtz-Meydan et al., 2018; Ramos et al., 2018). As is the case with most human needs and impulses, sex is experienced within a normative framework that dictates what is proper and legitimate for different social groups (Wiederman, 2015; Gore-Gorszewska et al., 2023). Age, gender, and class -among others- are social markers that often determine what is considered acceptable, or even proper, in this domain. From this perspective, the idea of older people as asexual would stem mainly from socially constructed norms that portray them as passive, fragile or even infirm, an image that is not compatible with the common-sense view on “sexiness” (Dominguez and Barbagallo, 2016). Most importantly, such an idea does not have its origin in older people's experience, but in how other social groups -typically, younger ones- imagine life after retirement age (Snyder and Zweig, 2010; Thompson et al., 2014).

This article seeks to contribute to the existing knowledge on how people over 60 years of age -the threshold to be considered an older adult, according to the United Nations (2019)- see sexuality in this life stage, based on a Chilean sample. As Harding and Peel (2016) argue, aging sexualities are not only nuanced by age itself, but also their intersection with other social and cultural locations, including ethnicity, disability, class and of course, gender (Katz and Calasanti, 2015; Bozon et al., 2018; Gore-Gorszewska et al., 2023). Of these, we focus on the latter two, because of their importance as criteria for social stratification and distribution of life opportunities in Chile, one of the most aged societies in Latin America. As it will be discussed in the following pages, throughout the life cycle gender is a main normative framework governing romantic relationships and sexuality, establishing differentiated norms for men and women, often under strong heteronormative assumptions. In the same vein, in highly unequal societies -like Chile [Programa de las Naciones Unidas para el Desarrollo (PNUD), 2017]- class conditions access to information and resources that might be fundamental in shaping people's view on sexual practices and reproduction.

## 2 Sexuality and aging: an intersectional approach

A fundamental assumption of this study is that aging, although a biological process, has a key social component. The meanings attached to the physiological changes associated with aging vary according to definitions that are socially and historically situated, as do the social roles and behaviors accepted for each life stage. Like ethnicity and gender, age is inscribed in the body, so it can easily be forgotten that it is mainly this social interpretation and normativity (and not only bodily capabilities) that dictates what people can and cannot do (Twigg, 2004; Calasanti and King, 2018). Aging bodies can be regarded as aesthetically pleasant, prestigious, or wise or, on the contrary, as decadent and dependent, based on how societies interpret the traces left by the passage of time. Culture assigns to each age group a set of expectations about how they should behave, and what is appropriate (or not) for them. These expectations can easily become prejudices about what it means to belong to a certain age group: that is, what identified as ageism (Ayalon and Tesch-Römer, 2018). The concept refers to the stereotyped view on the characteristics and capabilities of a certain age group that might influence not only the way in which society sees members of such a group, but also how individuals in the group see themselves (Ayalon and Tesch-Römer, 2018).

Ageism manifests itself in attitudes, behaviors, and even institutional arrangements. Gewirtz-Meydan et al. (2018) point out that, until recently, research on sexuality paid scarce attention to the sex lives of older adults, reflecting the preconception that this was not a matter worthy of much academic scrutiny. The issue was the subject of perusal from the health sciences, although often in a way that focused on health problems preventing the elderly from having an active and satisfactory sex life (DeLamater and Koepsel, 2015). Distancing oneself from ageism implies acknowledging that no age group is homogeneous: social locations such as class and gender play a significant role in shaping individuals' life experiences, and sexuality is no exception (Gore-Gorszewska et al., 2023). As intersectionality theory suggests, these social categories -age, gender and class- do not simply add to one another but overlap in order to position individuals differently regarding social hierarchies (Hill and Bilge, 2016). Intersectionality is tied to social structures that distribute life opportunities -in this case, to express oneself in the realm of sexuality- and chances of being subjected to a tighter control of sexual behavior, rendered invisible as a sexual being or even oppressed. Therefore, each form or oppression is dependent upon and shapes the other.

Intersectionality provides some useful insights: on the age front, older people's sexuality is often made invisible: eroticism and desire are posed in opposition to aging, establishing an image of old age that is characterized by erotophobia and the restriction of sexual pleasure in this life stage (Bozon et al., 2018).

At the same time, this denial of sexual capabilities does not occur in the same way for men and women. Gender is a highly normative framework that governs sexual conduct in individuals of all ages (Bajos and Bozon, 2012; Wiederman, 2015; Gore-Gorszewska et al., 2023). Usually, normative assumptions depict male sexuality as guided by impulse and associated with pleasure, whereas women's is linked to emotion and motherhood (Calasanti and King, 2018). The traditional, heteronormative construction of masculinity and femininity has often implied a dichotomous view in which men were supposed to be assertive, dominant, and with a high sex drive. Women, on the contrary, were expected to be able to control their sexual impulses, limit their expression to emotionally committed relationships and let their (male) partners take the lead in sexual matters. As people age, it is assumed the sex drive will weaken or even disappear, particularly in women (Twigg, 2004). Paradoxically, although older individuals are perceived as somehow de-sexualized, gender still plays a part in how their sexuality is characterized (Calasanti and King, 2018). For instance, given the strong cultural association between female sexuality and motherhood, the end of reproductive years for women -marked by menopause- is often accompanied by the idea that women's interest in sex will greatly diminish after they can no longer become pregnant. This normative framework allows men to express their sexuality more freely for longer, without social disapproval (Lai and Hynie, 2011).

Likewise, class plays a role in shaping the way in which individuals conceive of gender relations, sexuality and reproduction (Træen et al., 2018). The literature suggests that individuals with lower levels of income and formal education tend to adhere to more traditional views on gender, assuming that sexuality and reproductive behavior are governed mainly by biology -thus downplaying the role of social norms. They also tend to give less relevance to the aspects of communication, affectivity, and emotional intimacy in sexual relationships (Higgins and Browne, 2008; Træen et al., 2018). Therefore, this group is more likely to hold stereotypes on both gender and age that will affect their own chances of remaining sexually active throughout the life cycle.

Conversely, people with higher levels of income and education tend to be more critical of traditional gender arrangements, assigning more importance to the social norms governing sexuality than to biology to explain gender differences. This group is also more likely to engage in a wider range of sexual practices (Higgins and Browne, 2008) and to regard sexuality as part of their self-expression, which implies a more flexible reading of social norms so as to accommodate one's own desires and preferences (Higgins and Browne, 2008). Higher income and education make it possible for individuals to count on more material and symbolic resources -for instance, a wider gamut of ideas on sex and sexual relationships- that give them more bargaining power to discuss relationships and family arrangements with a partner. This association has been studied in young people (AUTHOR, blinded for review) but comparatively much less attention has been given to such relations in older adults (Brown and Shinohara, 2013).

### 2.1 Aging and sexuality in Chile

In many societies -including Chile- what is socially considered “old age” starts with retirement and is characterized as a point in which people's physical and mental capabilities begin to decline. According to recent data (Universidad Católica de Chile, 2021) Chileans consider that old age starts at 65–66, although the legal retirement age is 60 for women and 65 for men. As we mentioned earlier, Chile is one of the countries with a higher proportion of adults over 60 years of age in Latin America, along with Uruguay and Cuba: the most recent data available show that in 2017 they comprised 19% of the population (Ministerio de Desarrollo Social y Familia de Chile., 2017; Pavez Lizarraga et al., 2023). Nationwide data on sexuality in this age group is relatively scarce; nonetheless, a 2019 study (Universidad Católica de Chile - Caja los Andes., 2019) shows that 60% consider sexuality a key part of life. Out of that figure, 22% think that sex life is equally important in older age as it was in their younger years, whereas the rest (38%) considers that its importance diminishes with age. Among people aged 60–69, men with high levels of formal education and steady partners are the subgroup that declares to have more active sex lives (Universidad Católica de Chile - Caja los Andes., 2019; Universidad Católica y Caja Los Andes, 2023). Although the Chilean State has implemented policies aimed at improving older people's life opportunities in health and leisure activities, sexuality is usually overlooked as an issue for public policy, despite the evidence showing its importance for this age group (Pavez Lizarraga et al., 2023). Surveys carried out in the US and European countries confirm that sexuality changes over the life course, but it does not lose centrality in people's lives. The University of Michigan National Poll on Healthy Aging, which asked a national sample of US adults aged 65–80 about their perspectives on relationships and sex, reports that most older adults (76%) agreed that sex is an important part of a romantic relationship at any age. Men were more likely to agree (84%) than women (69%). Two in five (40%) indicated that they were sexually active at the time the survey was applied (2017) and about 65% of the respondents described being interested in sex (30% extremely or very interested, 35% somewhat interested). As a tendency, men and younger groups (65–70, compared to 70 and over) reported to be more interested in sex and sexually active in a higher proportion (University of Michigan, 2018). In a similar vein, the English Longitudinal Study of Aging showed that a sizable portion of middle-aged and older men and women remain sexually active, even until the eighth decade of life (Lee et al., 2016). This research, among other efforts, confirms previous nationwide studies that confirmed that people remain sexually active well into old age and even in the presence of cognitive impairment (Waite et al., 2022).

## 3 Materials and methods

### 3.1 Participants and procedure

Participants in this study were adults aged 60 and over. The study relied on computer assisted telephone interviews (CATI), a surveying technique where interviews are carried out via a phone call with the interviewer using one electronic device (computer/smartphone/tablet) to both read the survey script and enter the information collected. This method was selected for its capacity to provide a wider reach, including older people from different geographical origins and those who had limited opportunities to be in public spaces, a requirement for face-to-face data collection. The sample was obtained through Random Digit Dialing (RDD), using a nationwide database provided by an external consultant. A total of 481 valid interviews were obtained; representation considered two sexes (51% male, 49% female) and socio-economic level, as a proxy for class. The latter was operationalized following the European Society for Opinion and Marketing Research (ESOMAR) model, which considers the profession or occupation of the respondent and his/her educational level. It goes from ABC1 (Very high-high), C2–C3 (middle), D (middle-low to low) to E (very low). The data were collected by Cadem, a company specialized in market and opinion research (http://cadem.cl). Approval from an Ethics Committee was not required, since the company's protocols were already approved by the Universidad Autónoma, institution that funded the data collection trough an internal competition called “Bitácora Social”. All the respondents had to explicitly give their consent to proceed with the interview. Average age was 69.1 ± 6.6 years.

### 3.2 Instruments and analysis plan

The questionnaire was elaborated selecting and adapting items from two existing, validated instruments: the National Study of Sexual Behavior (*Encuesta Nacional de Comportamiento Sexual*, COSECON), applied in Chile in 1998 by the Ministry of Health (Ministerio de Salud de Chile., 2000) in association with the French Agence Nationale de Recherche sur le SIDA (ANRS). Up to the moment this article is written, this is the only comprehensive study on sexuality in Chile^1^, although surveys with subgroups of the population (mainly young people) are carried out on a regular basis. For this reason, we decided to replicate some items of this measure, since these surveys could provide a baseline for the age group we are analyzing. The second instrument is the report (Bajos and Bozon, 2008) made of the survey Context of Sexuality in France, applied in 2006 to individuals aged 18–69. The final questionnaire used in this study consisted of 15 items, including socio-demographics questions, three dichotomous (yes/no) items and five Likert-type scales of five points, ranging from “Not acceptable at all” (1) to “Entirely acceptable” (5). Despite a growing body of literature addressing the topic, there is still no consensus on how to measure older adults' sexuality, which might present some differences when compared to younger groups (Cameron and Santos-Iglesias, 2024). As the survey was designed to be carried out telephonically (as opposed to anonymous self-report), we selected items that were as non-invasive as possible, to increase our chances of obtaining a good response rate for the complete interview. Data were collected during the COVID-19 pandemic and in the aftermath of months of social unrest after the October 2019 social protests in Chile, which made other interviewing methods unfeasible.

Data were analyzed using descriptive and inferential statistics; the latter group considered bivariate and multivariate analysis to establish possible differences according to sex and socio-economic level. All the statistical tests have a significance level of 5%, and were carried out using Jasp, Jamovi and SPSS (v.28) software.

## 4 Results

Most interviewees in our sample (93%) were in a long-term relationship at the moment the survey was applied: 62% declared to have been with the same partner for the last 40 years, and only a small percentage (3%) had been with their current partner for less than 10 years. Comparatively, the percentage of older people currently in a relationship is higher than the figure reported by national studies not focused on sexuality, which is about 50% (Universidad Católica y Caja Los Andes, 2023). Other surveys (Ministerio de Desarrollo Social y Familia de Chile, 2023) indicated that around 8% of individuals aged 60 or over report a break up in the last 5 years. Mean age for the first marriage or steady relationship with cohabitation was 27 years for men and 25 for women. Consulted about the propriety of starting a romantic relationship after 60, 87% considered it acceptable or very acceptable, without statistically significant differences by sex or socio-economic level. Nonetheless, consulted specifically about the importance of sex in this life stage, result vary, as Table 1 shows.

**Table 1:** Perceptions on sexuality in older adults by sex- chi-square tests.

**Item**	**Category**	**Sex**		**Total% [*n* = 481]**	**χ^2^**	**df**	**p**	**Size eff**.
		**M% [*****n*** = **244]**	**W% [*****n*** = **237]**					
1. Do you think it is important that people over 60 years of age have an active sex life?	*Yes*	**93**	**78**	**85**	χ^2^ = 22.4	2	<0.0001	0.22
	*No*	4	16	10				
	*No answer*	3	7	5				
2. What do you think about a couple in which the man is older (let's say, 10 years of more) that the woman?	*Not at all/not quite acceptable*	10	10	10	χ^2^ = 1.13	2	0.57	0.05
	*Maybe acceptable*	14	18	16				
	*Acceptable or highly acceptable*	**76**	**72**	**74**				
3. What do you think about a couple in which the woman is older (let's say, 10 years of more) that the man?	*Not at all/not quite acceptable*	18%	20	19	χ^2^ = 0.66	2	0.72	0.03
	*Maybe acceptable*	17	18	18				
	*Acceptable or highly acceptable*	**65**	**62**	**64**				
4. What do you think about older people having an active sex life without intention of establishing a long-term relationship?	*Not at all/not quite acceptable*	11	24	18	χ^2^ = 16.1	2	<0.001	0.19
	*Maybe acceptable*	20	13	17				
	*Acceptable or highly acceptable*	**68**	**63**	**66**				
5. What do you think about a man over 60 paying for sex?	*Not at all/not quite acceptable*	**56**	**61**	**59**	χ^2^ = 1.83	2	0.4	0.06
	*Maybe acceptable*	12	13	12				
	*Acceptable or highly acceptable*	32%	26	29				
6. What do you think about a woman over 60 paying for sex?	*Not at all/not quite acceptable*	**59**	**71**	**65**	χ^2^ = 9.12	2	0.01	0.14
	*Maybe acceptable*	14	13	13				
	*Acceptable or highly acceptable*	27%	16	22				
7. Do you think the lack of sex life is a proper reason to end a marriage or long-term relationship?	*Yes*	**53**	43	48	χ^2^ = 5.85	2	0.05	0.11
	*No*	44	**55**	**50**				
	*No answer*	3	2	3				
8. Do you think there is an age limit to enjoy sex?	*Yes*	13	16	15	χ^2^ = 1.63	2	0.44	0.06
	*No*	**86**	**82**	**84**				
	*No answer*	1	2	1				

Values in bold indicate answers with the highest percentage.

As it can be seen in Table 1, most participants (85%) consider it important for older adults to remain sexually active, with statistically significant differences by sex: men declare it to be so in higher proportion than women (93% vs. 78%). This figure is consistent with international studies, mainly in the US and Europe, that situate this figure around 75% (Portellos et al., 2023; Steckenrider, 2023), although figures vary greatly depending on how sexuality is operationalized (Cameron and Santos-Iglesias, 2024). According to a 2023 national survey (Universidad Católica y Caja Los Andes, 2023), a third of people aged 60 and over in Chile declare to be sexually active. The same survey shows that ceasing sexual activity (operationalized as not having sex in a year or more) after 60 is more common for women (48%) than for men (26%) -again, a finding that is consistent with the existing literature on the topic (University of Michigan, 2018; Goetschy et al., 2022; Cameron and Santos-Iglesias, 2024). Regarding sizable age differences within couples (ten years or more), both sexes consider them acceptable, although a situation in which men are older than their partners obtained higher acceptance (74%) than the opposite case (younger men with older women, 64%). The latter situation, albeit becoming more socially unobjectionable (Vanderheiden, 2021), is still more frowned upon than age gaps in men's favor, presumably because male sexuality is seen as less affected by age. The cultural association between female sexuality and motherhood implies that women are not seen as sexual beings after the end of their reproductive years (Pino Alvarez et al., 2022).

For Question four, 66% of participants considered “Entirely acceptable” or “ Acceptable” that older people might maintain an active sexual life without the intention of forming a steady romantic partnership. Results for question four show differences by gender (*p* < 0.001), mainly because 24% of women disapprove such a situation (“Not acceptable at all” or “Not quite acceptable”), whereas 20% of men think of it as maybe acceptable. This suggests that the idea that “proper” female sexuality should be displayed only in the context of emotionally committed relationships still holds some validity in this group. Likewise, albeit 59% disapproves of older men paying for sex (Question five), the proportion increases to 65% in the case of older women doing the same (Question six), and women disapprove more (71%) than men (59%), with significant differences (*p* = 0.01).

Opinions are divided regarding the validity of lack of sexual life as a reason to end a marriage or stable relationship (Question seven), with 53% of men and 43% of women declaring that this is a compelling reason. The difference is statistically significant (*p* = 0.05). This suggests that sex -as opposed to companionship, which according to the stereotype is what older people would look for in a relationship- is more relevant for men than for women. However, 84% of the respondents agreed with the statement that there is no age limit for enjoying sex (Question eight), with no differences between men and women.

From a class perspective, similar differences can also be found, as Table 2 shows. For Question one, between 94% (ABC1) and 81% (D) declared that it is important to maintain an active sex life after turning 60; however, this figure drops to 60% in the groups with less income and education (*p* < 0.001, Cramer's V = 0.18). As in the case of gender, higher-income groups consider age differences more acceptable, particularly when men are the older party. Lower income and education groups give less support to this idea: only 43% of the respondents in the E group considers it acceptable when women are older than their partners, with significant differences only for question 3 (*p* = 0.03, Cramer's V = 0.13). Such a result suggests that less educated individuals tend to endorse more traditional sexual norms, including the double standard that often goes with it (Vanderheiden, 2021). Again, paying for sex is not considered appropriate for older adults, but much less so in the case of women (29% in Question 5 vs. 22% in Question 6).

**Table 2:** Perceptions on sexuality in older adults by socio economic level- chi-square tests.

**Item**		**Socio Economic Level (SEL)**					**Total% [*n* = 481]**	**χ^2^**	**df**	**p**	**Size eff**.
		**ABC1% [*****n*** = **94]**	**C2% [*****n*** = **126]**	**C3% [*****n*** = **111]**	**D% [*****n*** = **120]**	**E% [*****n*** = **30]**					
1. Do you think it is important that people over 60 years of age have an active sex life?	*Yes*	**94**	**89**	**86**	**81**	**60**	**85**	χ^2^ = 30.6	8	<0.001	0.18
	*No*	3	6	11	15	20	10				
	*No answer*	3	5	4	4	20	5				
2. What do you think about a couple in which the man is older (let's say, 10 years or more) than the woman?	*Not at all/not quite acceptable*	6	6	9	17	13	10	χ^2^ = 14.1	8	0.08	0.12
	*Maybe acceptable*	14	17	20	12	20	16				
	*Acceptable or highly acceptable*	**80**	**77**	**71**	**71**	**67**	**74**				
3. What do you think about a couple in which the woman is older (let's say, 10 years or more) than the man?	*Not at all/not quite acceptable*	15	15	21	18	40	19	χ^2^ = 17.0	8	0.03	0.13
	*Maybe acceptable*	14	24	17	15	17	18				
	*Acceptable or highly acceptable*	**71**	**61**	**62**	**68**	**43**	**64**				
4. What do you think about older people having an active sex life without the intention of establishing a long-term relationship?	*Not at all/not quite acceptable*	15	12	18	25	17	18	χ^2^ = 12.1	8	0.15	0.11
	*Maybe acceptable*	14	19	12	21	17	17				
	*Acceptable or highly acceptable*	**71**	**69**	**70**	**55**	**67**	**66**				
5. What do you think about a man over 60 paying for sex?	*Not at all/not quite acceptable*	**59**	**59**	**58**	**62**	**43**	**59**	χ^2^ = 7.12	8	0.52	0.09
	*Maybe acceptable*	14	14	12	7	21	12				
	*Acceptable or highly acceptable*	27	27	30	31	36	29				
6. What do you think about a woman over 60 paying for sex?	*Not at all/not quite acceptable*	**61**	**65**	**65**	**70**	**57**	χ^2^ = 3.48	8	0.9	0.06	
	*Maybe acceptable*	15	13	15	10	14	13				
	*Acceptable or highly acceptable*	24	22	20	19	29	22				
7. Do you think the lack of sex life is a proper reason to end a marriage or long-term relationship?	*Yes*	36	44	49	**57**	**63**	48	χ^2^ = 22.2	8	0.01	0.15
	*No*	**61**	**55**	**50**	42	27	**50**				
	*No answer*	3	2	2	2	10	3				
8. Do you think there is an age limit to enjoy sex?	*Yes*	6	6	11	27	43	15	χ^2^ = 54.0	8	<0.001	0.24
	*No*	**93**	**94**	**87**	**73**	**53**	**84**				
	*No answer*	1	0	3	1	3	1				

Values in bold indicate answers with the highest percentage.

For Question seven, higher income groups disagree more with the idea that lack of sex life is a valid reason to put an end to a marriage or steady relationship −61% (ABC1), 55% (C2) and 50% (C3)- than their lower income counterparts (42% in D and 27% in E), with significant differences (*p* = 0.005, V = 0.15). This result is consistent with the hypothesis that less educated groups tend to downplay the aspects of communication, shared interests, and emotional intimacy present in sexual relationships (Higgins and Browne, 2008; Træen et al., 2018) -so when physical intimacy is over, the relationship itself might be ended. For upper-class individuals, ending a marriage or long-term relationship also might pose concerns about other issues, like the division of property and the impacts on the “investment” made in children that come with separation or divorce (Lundberg and Pollak, 2015).

In a similar vein, in Question eight the possibility of enjoying sex at any age is supported by a vast majority in the ABC1 (93%), C2 (94%) and C3 groups (87%) but drops significantly in the D (73%) and E groups (53%) (p = 0.005, V = 0.15). Besides the considerations mentioned above, to account for this difference it is worth mentioning that in Chile upper-class individuals tend to grow old in much better health conditions than lower-income elderly (Universidad Católica de Chile - Caja los Andes., 2019; Universidad Católica y Caja Los Andes, 2023), which might improve their prospects about having an enjoyable sex life for longer.

In a later stage of analysis and to assess the relative weight of each variable, binary logistic regression analysis was used for the questions that produced significant results for both gender and socio-economic level. These analyses were carried out for Question one, “Do you consider it important that people over 60 years maintain an active sex life?”, Question seven, “Do you think that lack of sex life is a valid reason to end a marriage or steady romantic partnership?” and Question eight, “Do you think there is an age limit to enjoy sex?”. The results are shown in Tables 3–5.

**Table 3:** Do you consider important that people over 60 years maintain an active sex life?—Binary regression.

**Predictor**	**Model 1**	**Model 2**	**Model 3**
Age	0.96 (0.91–0.99)^*^	0.96 (0.92–1.01)	0.96 (0.92–1.01)
Sex (Women)	–	0.23 (0.11–0.47)^***^	0.24 (0.11–0.49)^***^
SEL (ABC1)	–	–	9.24 (2.04–41.8)^**^
SEL (C2)	–	–	4.41 (1.32–14.8)^*^
SEL (C3)	–	–	2.65 (0.84–8.32)
SEL (E)	–	–	1.88 (0.63–5.64)
**Goodness-of-fit statistics**			
χ^2^	4.36	23.61	36.95
df	1	2	6
Significance	0.037	<0.001	<0.001
R^2^N	0.02	0.10	0.16
R^2^CS	0.01	0.05	0.08
R^2^MF	0.01	0.08	0.12
Δχ^2^		19.2	13.3
Significance		<0.001	0.01
**Prediction (cut-off point** = **0.90)**			
Precision	61%	64%	74%
Specificity (No)	53%	75%	68%
Sensitivity (Yes)	62%	62%	75%

Asterisk indicates statistically significant results.

**Table 4:** Do you think that lack of sex life is a valid reason to end a marriage or steady romantic partnership?

**Predictor**	**Model 1**	**Model 2**	**Model 3**
Age	1.00 (0.97–1.03)	1.00 (0.97–1.03)	0.99 (0.97–1.03)
Sex (Mujer)	–	0.65 (0.45–0.93)^**^	0.60 (0.41–0.87)^**^
SEL (ABC1)	–	–	0.23 (0.09–0.58)^**^
SEL (C2)	–	–	0.31 (0.126–0.77)^*^
SEL (C3)	–	–	0.39 (0.16–0.98)^*^
SEL (D)	–	–	0.55 (0.22–1.36)
**Goodness-of-fit**			
χ^2^	0.001	5.59	21.91
df	1	2	6
Significance	0.974	0	0.001
R^2^N	0.00	0.02	0.06
R^2^CS	0.00	0.01	0.05
R^2^MF	0.00	0.01	0.03
Δχ^2^		5.59	16.3
Significance		0.018	0.003
**Prediction (cut-off point** = **0.90)**			
Precision		55%	61%
Specificity (No)		55%	54%
Sensitivity (Yes)		56%	67%

^*^indicates statistically significant results. ^**^ indicates statistically significant results in two-tailed tests.

**Table 5:** Do you think there is an age limit to enjoy sex?

**Predictor**	**Model 1**	**Model 2**	**Model 3**
Age	1.00 (0.97–1.03)	1.00 (0.97–1.03)	0.97 (0.94–1.01)
Sex (Women)	–	0.65 (0.45–0.93)^**^	0.88 (0.51–1.52)
SEL (ABC1)	–	–	11.7 (3.89–35.5)^***^
SEL (C2)	–	–	13.8 (4.79–39.9)^***^
SEL (C3)	–	–	6.63 (1.56–17.2)^***^
SEL (D)	–	–	2.26 (1.03–5.25)^*^
**Goodness-of-fit**			
χ^2^	2.49	3.2	47.06
df	1	2	6
Significance	0.115	0.201	<0.001
R^2^N	0.01	0.01	0.17
R^2^CS	0.01	0.01	0.09
R^2^MF	0.01	0.01	0.12
Δχ^2^		0.72	43.9
Significance		0.397	<0.001
**Prediction (cut-off point** = **0.90)**			
Precision		61%	69%
Specificity (No)		46%	71%
Sensitivity (Yes)		64%	68%

Asterisk indicates statistically significant results.

As Table 3 shows, it was found that the likelihood of considering important that older adults have an active sex life is associated with sex, with women having lower odds (*p* < 0.001, OR = 0.24, 95%CI = 0.11–0.49). In socio-economic terms, belonging to ABC1 group is associated with a higher likelihood than being in the D group (*p* < 0.01, OR = 9.2, 95%CI = 2.04–41.8), and C2 has a higher likelihood than E *p* < 0.05, OR = 4.4, 95%CI = 1.32–14.8).

Results on Table 4 confirm that the probabilities of considering the lack of sex as a reason for ending a relationship are associated with sex (52%), being lower for women (46% vs 59% for men, *p* < 0.001, OR = 0.60, 95%CI = 0.41–0.87) and with socio-economic level, since the ABC1 group is less likely (36%) to give an affirmative answer than E (72%) (*p* < 0.01, OR = 0.23, 95%CI = 0.09–0.58), C2 (44%) (*p* < 0.05, OR = 0.31, 95%CI = 0.13–0.77), C3 (50%), and D (58%) (*p* < 0.05, OR = 0.39, 95%CI = 0.16–0.98).

In a similar vein, results on Table 5 show that there are no gender differences concerning the belief that there is an age limit to enjoy sex, with similar probabilities for men (86% men, 85% women) to disagree with such a statement (*p* = 0.66, OR = 0.88, 95%CI = 0.51–1.52), but in terms of socio-economic level, the lower income group (E) has significant more chances of choosing the affirmative answer (45%) than any of the other groups (ABC1 = 6%, C2 = 5%, C3 = 11%, D = 26%) (*p* < 0.05).

## 5 Discussion

Data examined in these pages shows that Chilean older adults, both men and women, see sexuality as a key part of their lives. This is consistent with international evidence from studies in the United States (McWilliams and Barrett, 2012; Brown and Shinohara, 2013), Germany (Schlagdenhauffen, 2011), France (Bozon et al., 2018), Spain (Bretin and Gómez-Bueno, 2011), United Kingdom and Australia (Portellos et al., 2023), indicating that the renewed interest in older sexualities would be akin to a new “sexual revolution” (Curley and Johnson, 2022). Bozon (2013) points out that this may lead to a new norm: to be in a relationship is to have a sexual life, at any age. According to Gore-Gorszewska et al. (2023), generally speaking older adults in long-term intimate relationships benefit from the established intimacy and engage in partnered sex well into later life because it strengthens their bond, enhances their love, and conveys commitment. Contrary to common-sense perception, desire and the sex drive do not necessarily fade with the years, albeit their expression changes throughout the life cycle (Schlagdenhauffen, 2011; Bozon, 2013; Dominguez and Barbagallo, 2016). Our results suggest that gender and class also play a key role in influencing the way older adults see their relationship with sexuality, although the percentage of individuals holding a romantic partnership (higher than the national average) might tint this perception. Men and the more educated, affluent groups tend to have a more positive view on sex at later stages in life, distancing themselves from the stereotype according to which sex is a matter for the young, which is consistent with government data on the matter (Universidad Católica de Chile - Caja los Andes., 2019; Universidad Católica y Caja Los Andes, 2023). Also, in their systematic review of the literature on the issue, Cameron and Santos-Iglesias (2024) reported three main findings: (1) that large percentages of older adults continued to have sexual activity into old age, (2) that older women were less sexual activity than male older adults, and (3) that sexual activity declines with increasing age of participants, with the 60–69 groups being more active than the 70 and over adults.

In line with intersectionality theory, some gendered norms about sexuality still are held as valid in this age group, such as sex being more important for men, who face less prejudices regarding having partners that are much younger than themselves, paying for sex or having sexual encounters outside emotionally committed relationships. From this perspective, upper class women that are 60 or over would have more possibilities of expressing their sexuality than their less affluent counterparts, but fewer than men in their own class. Societies are typically more restrictive with the expression of female desire and sexual impulses, but after the end of the reproductive years, female sexuality is often rendered invisible. As Sinković and Towler (2019) observe, women face a double burden of ageism and sexism. The stereotype of asexual old age seems to apply more to women than men (Bradway and Beard, 2015). Similarly, Calasanti and King (2018) point out that strong, dominant, and assertive (hetero)sexuality is a key element in the construction of the so-called “hegemonic masculinity”. Therefore, for men declaring to be able to maintain sexual activity might be a marker of prestige and power from the viewpoint of the traditional forms of male identity, which places other expressions of masculinity in a subordinate place. Thus, keeping themselves sexually active would be crucial for men not only from the perspective of the satisfaction of sexual and affective needs, but also as an assertion of what it means to be a man. Our data also contributes to the growing body of literature that provides evidence that older adults, men and women, openly acknowledge the importance of sexuality, although still under the gendered assumption that it is more relevant for men.

In a similar vein, the differences found by socio-economic level provide evidence to support the hypothesis that is cultural and social conditions -not biology only- that are crucial to allow older people to have an enjoyable sex life (Schlagdenhauffen, 2011). Higher levels of education and income supply individuals with key symbolic resources -access to better information, more critical views on religious and gendered social norms- to deal with the life challenges that aging might bring; but also with material conditions, such as better health care and nutrition and a place where they can have privacy, to mention some, that favor physical intimacy (Gore-Gorszewska et al., 2023). Socio-economic differences found in this study most likely reflect life trajectories marked by unequal access to educational, health and financial resources that impact on people's experiences in the sexual domain. This finding corroborates previous research suggesting that educational and economic resources are a key part of older people's experience of sexuality (Ortega González, 2018; Undurraga et al., 2019). The lack of steady data production regarding older people's sexuality makes it difficult to explore this hypothesis in greater depth, as it does the lack of consistent measurements across the literature (Cameron and Santos-Iglesias, 2024).

Notwithstanding, as Træen et al. (2018) observe, the sexual culture and gendered roles dominant in a society might exert a considerable influence on how freely individuals can express their needs and preferences in this domain. Thus, these findings have at least two relevant implications: on the one hand, an inclusive, more egalitarian approach to sexuality should be included among public policy's goals to foster older people's wellbeing. As State policy and programs are often aimed at individuals with lower economic and educational resources, it is crucial that these do not reproduce ageist and gendered views. For instance, health programs defined as women's health often focus on reproductive needs, often overlooking the fact that women might need information and services on sexual health beyond the childbearing years. There is some evidence supporting the notion that a non-ageist environment that acknowledges broader definitions of sexuality and the importance of pleasure at all ages might increase older persons' interest in remaining sexually active (Faus-Bertomeu and Gómez-Redondo, 2017). On the other, as individuals aged 60 or over do not need to be concerned about unwanted pregnancies, they might also minimize the health risks of having a sexually active life, including sexually transmitted diseases (STDs) (Khan et al., 2023; Pavez Lizarraga et al., 2023). Educational efforts usually target mainly younger populations, often under the premise that older people are more risk-aware or no longer at risk at all, since they either do not have sexual activity or have it in the context of a monogamous relationship. The data suggest that this might not be the case (Goetschy et al., 2022). Sexuality encompasses self, interactions with others, and various stages of expression and affection throughout life (Dominguez and Barbagallo, 2016).

## 6 Study limitations

The present study presents only perceptions about sexuality from men and women over 60 years of age, which provides a glimpse on how this age group sees norms and beliefs about sexuality. We did not ask about actual practices, which is a limitation of this study. We understand that the instrument might have a limited scope, as we focused on questions people felt comfortable answering on the phone. However, the data presented contributes to a better understanding of a relatively under researched age group and, most importantly, to shed light on how social stratification criteria, like socio-economic level and gender, intersect with age to shape individuals' opportunities to explore and express their own sexuality. We also understand the limitations of the ESOMAR model as a proxy for class (Joignant and Güell, 2009) especially for older people, but, given its ample use on both marketing research and government surveys, it allows us to have information that can be compared with government data in future research. It is important to include older people's concerns in surveys on sexuality, such as the availability of services and pertinent information on sexual health and the possibility of having adequate places to enjoy physical intimacy. The issue is further complicated by the lack of a standardized measurement of sexual activity in older adulthood, which would allow researchers to better grasp when/if societal factors are influencing one culture's sexual activity compared to another (Cameron and Santos-Iglesias, 2024). As Cameron and Santos-Iglesias (2024) point out, the way an older adult's sexuality expresses itself -and how this age group think of sexuality- might be different from people of reproductive age, and this needs to be considered in future research.

## 7 Conclusion

Sexuality in old age is still conditioned by biases, prejudices, and a stereotyped vision, despite several studies and surveys showing that older persons have an undeniable sexual potential to express (Bessin and Blidon, 2011; Dominguez and Barbagallo, 2016). This ageist view also intersects with social markers like gender and class, which means that older people -like any other age group- will not have homogeneous perceptions and experiences in the sexual domain. As our data show, income and education are key in shaping the way in which people see their own sexual capabilities in older age. Likewise, gender is a normative framework that establishes what is “proper” and what is not throughout the life cycle, and it is usually more restrictive for women, even beyond the reproductive years.

Bearing this in mind is relevant to avoid not only the de-sexualization of people after retirement age but also to highlight that the opportunities of enjoying sex in older years are unequally distributed. This implies that experiences, perceptions, and needs will be different, and this must be taken into account by public policies aimed at this age group. Such policies often assume individuals can choose certain lifestyles (Katz and Calasanti, 2015) when in reality they are constrained by social and cultural factors other than just age, as intersectionality theory argues. In doing so, they obscure the role gender and class (among other social markers not examined in this study, such as ethnicity) play in allowing or denying access to material and symbolic resources throughout life -resources that are crucial for a more unconstrained expression of sexuality (Katz and Calasanti, 2015). These social cleavages are also relevant in influencing the meaning older people attribute to romantic partnerships in the so-called third age (60–65 years old) (Brown and Shinohara, 2013). In this case, access to health services in general–and sexual health services in particular–and the availability of adequate spaces for socializing could be important for the development of older people's interests and capacities around sexuality (Pino Alvarez et al., 2022), which have not been addressed by the Chilean State.

The changing demographic context, with the lengthening of life expectancy, invites us to think about the articulation between aging, gender and sexuality. This has effects at the level of health planning, e.g., the development of policies aimed at the health and wellbeing of older people. One of the most obvious problems older people face is the lack of access to health services, particularly sexual health services. This point is crucial because, in addition to the cultural barriers to the patients to consult on these issues, it is the lack of preparation of health professionals to deal with their consultations (Ezhova et al., 2020). At the policy level, while there is a growing awareness of the need to deepen and improve policies toward the aging population, these have not yet been fully modernized. For example, in 2017 Chile ratified the Inter-American Convention on the Protection of the Human Rights of Older Persons, which refers to the commitment to formulate public policies, plans and strategies that in turn must be created, implemented and evaluated to promote active and healthy aging (Mora and Herrera, 2018). However, to date, an incomplete enshrinement of these rights has been detected (Miranda, 2018), as Chilean legislation lacks norms that specifically protect the sexual health of older adults and/or their right to privacy. Chile is no exception in the dispersion of the legal and institutional approach to protect its older adults, especially in rights that are considered “new” in legislation, and that have to do with emerging issues in public agendas, such as sexuality. In this context, few countries have incorporated concern for the sexual health of older adults into their plans, even though the importance of sexual wellbeing and sexual activity, and how these may impact mental health, has been recognized for a decade (Field et al., 2013).

## Data availability statement

The datasets presented in this article are not readily available because funding requires a period of embargo. Requests to access the datasets should be directed to gomezver@gmail.com.

## Ethics statement

Ethical approval was not required for the studies involving humans because methodology used a telephonic anonymous interview, oral consent was given, but not in written form. The studies were conducted in accordance with the local legislation and institutional requirements. Written informed consent for participation was not required from the participants or the participants' legal guardians/next of kin in accordance with the national legislation and institutional requirements because interviews were made on the phone, so only verbal consent was obtained.

## Author contributions

VG-U: Conceptualization, Writing – original draft, Investigation, Project administration. AG: Conceptualization, Funding acquisition, Writing – review & editing, Data curation. FT-N: Conceptualization, Methodology, Project administration, Supervision, Writing – review & editing.

## Footnotes

^1^Partial results from the National Survey on Health, Sexuality and Gender (Encuesta Nacional de Salud, Sexualidad y Género, ENSSEX) were released on October 13th, 2023. However, the results made available publicly do not differentiate by age groups, and the complete dataset is not available yet.
